# Antiviral Drugs and Vaccines for Omicron Variant: A Focused Review

**DOI:** 10.1155/2023/6695533

**Published:** 2023-09-08

**Authors:** Nidhi Garg, Ananya Sree Kunamneni, Pankaj Garg, Sandeep Sharma, Divakar Sharma, Adinarayana Kunamneni

**Affiliations:** ^1^Division of Infectious Diseases, Department of Internal Medicine, Mayo Clinic, Jacksonville, Florida, USA; ^2^Stanton College Preparatory School, Jacksonville, Florida, USA; ^3^Department of Chemistry, GLA University, Mathura, India; ^4^Department of Medical Laboratory Science, Lovely Professional University, Phagwara, Punjab 144411, India; ^5^Department of Microbiology, Lady Hardinge Medical College, New Delhi 110001, India

## Abstract

The Omicron variant of concern (VOC) replaced the delta variant rapidly and became the predominant strain due to more mutations in spike protein and receptor-binding domain (RBD) enhancing its infectivity and binding affinity. The severity of the illness is less than that of the delta variant. Omicron is nonsusceptible to REGEN-COV™ and bamlanivimab with etesevimab. Drugs that are effective against the Omicron variant are oral antiviral drugs such as Paxlovid (nirmatrelvir/ritonavir), remdesivir, sotrovimab, and molnupiravir. The potency of sotrovimab is reduced to 3-fold against Omicron, and 8-fold reduction in potency with sotrovimab is found in a particular variant of Omicron with a R346K substitution in spike protein. There are neither clinical trials comparing the efficacy of these 4 therapies with each other nor any data on a combination of two or more therapies. The current recommendation for mild-moderate, nonhospitalized patients who are at a high risk of disease progression is to use Paxlovid as the first-line option. If Paxlovid is not available or cannot be administered due to drug interactions, then the next best choice is sotrovimab. The third choice is remdesivir if sotrovimab is also not available and molnupiravir is to be given if the other three options are not available or cannot be administered. For prevention, 2130 (cilgavimab) in combination with COV2-2196 (tixagevimab) has been effective against BA.2 only. LY-CoV1404 (bebtelovimab) is recently authorized as it is effective against all sublineages of the Omicron variant. Regarding vaccine efficacy (VE), the 3-dose VE with mRNA vaccines at 14–60 days was found to be 71.6%, and after 60 days, it is 47.4%. There is a 34–38-fold reduction of neutralizing activity with prebooster sera and a 19-fold reduction with booster sera for the Omicron variant. This probably explains the reason for worldwide breakthrough infections with the Omicron variant with waning immunity. The neutralizing antibody response against Omicron elicited by the bivalent vaccine is superior to that of the ancestral Wuhan strain, without any safety concerns. For future advances, the ribosome display technology can be applied for the generation of human single-chain fragment variable (scFv) antibodies from B cells of recovered patients against Omicron and other Coronavirus variants as they are easier and faster to produce and have high affinity and high specificity.

## 1. Introduction

The Omicron variant (B.1.1.529) of Coronavirus is considered the variant of concern as its infectivity and transmissibility have strikingly increased to replace the delta variant and is now the predominant strain all across the globe. The estimated proportion of delta and other variants (B.1.617.2 and AY lineages) is 1.7% (95% PI 0.9–3%); however, of Omicron (B.1.1.529 and BA lineages) is 98.3% (95% PI 96.9–99.1%). Omicron has more than 30 mutations in the spike S protein, with 15 mutations located in the receptor-binding domain (RBD), crucial for virus to interact with the ACE-2 receptor. Virus infectivity is enhanced predominantly by cluster of mutations at the S1–S2 furin cleavage site, and binding affinity with human ACE-2 is increased due to mutations in RBD [1]. The Omicron variant has high environmental stability, high resistance against most therapeutic antibodies, and partial escape neutralization by antibodies from vaccinated individuals. Even though the severity of illness is milder than the delta variant, however, effective and specific treatment modalities are needed to prevent and treat illness to prevent morbidity. Omicron is nonsusceptible to REGEN-COV™ and bamlanivimab with etesevimab. Drugs that are effective against the Omicron variant are oral antiviral drugs such as Paxlovid (nirmatrelvir/ritonavir), remdesivir, S309 (sotrovimab), and molnupiravir. Sotrovimab is active against BA.1, but against BA.2, its activity dropped 27-fold. For prevention, 2130 (cilgavimab) in combination with COV2-2196 (tixagevimab) has been effective against BA.2 only. LY-CoV1404 (bebtelovimab) is recently authorized as it is effective against all sublineages of the Omicron variant [2]. With the rapid emergence of newer variants, it is essential to formulate novel strategies to curb the evolution of this virus. We propose a technology of short-chain fragment variable (ScFv) antibodies against Omicron and other emerging strains of Coronavirus as they are easier and faster to produce and also have higher specificity.

## 2. Mutations in Omicron Variant

Other than the 30 amino acid substitutions, three deletions, and one insertion, the Omicron variant's spike protein has mutations pertaining to its other genome parts as well. The three main sublineages of Omicron are B.1.1.529: BA.1, BA.2, and BA.3; they all share concerning substitutions in RBD. However, the specific substitutions in spike protein are specific to each lineage, e.g., H69 and V70 deletions are not seen in BA.2 lineage; hence, they do not have the spike gene target failure (SGTF) profile. These are the important substitutions in spike protein amino acids of various Omicron variants [3, 4]:

1. (B.1.1.529/BA.1):
2. A67V, del69–70, T95I, del142–144, Y145D, del211, L212I, ins214EPE, G339D, R346K, S371L, S373P, S375F, K417N, N440K, G446S, S477N, T478K, E484A, Q493R, G496S, Q498R, N501Y, Y505H, T547K, D614G, H655Y, N679K, P681H, N764K, D796Y, N856K, Q954H, N969K, and L981F
3. (B.1.1.529/BA.2):
4. T19I, delL24, delP25, delP26, A27S, G142D, V213G, G339D, S371F, S373P, S375F, T376A, D405N, R408S, K417N, N440K, S477N, T478K, E484A, Q493R, Q498R, N501Y, Y505H, D614G, H655Y, N679K, P681H, N764K, D796Y, Q954H, and N969K
5. (B.1.1.529/BA.3):
6. A67V, del69–70, del142–144, Y145D, del211, L212I, G339D, S371F, S373P, S375F, D405N, K417N, N440K, G446S, S477N, T478K, E484A, Q493R, Q498R, N501Y, Y505H, D614G, H655Y, N679K, P681H, N764K, D796Y, Q954H, and N969K

## 3. Differentiation between Delta Variant and Omicron Variant

The structural and clinical characteristics between two predominant strains, delta and Omicron, are shown in Table 1.

## 4. Therapeutics against Omicron

Unlike the delta variant, Omicron is nonsusceptible to REGEN-COV™ and bamlanivimab with etesevimab. Drugs which are effective against the Omicron variant are Paxlovid (nirmatrelvir/ritonavir), remdesivir, sotrovimab, and molnupiravir. The potency of sotrovimab is reduced 3–8-fold for a particular variant of Omicron with the R346K substitution in spike protein.

### 4.1. Ritonavir-Boosted Nirmatrelvir (Paxlovid)

Nirmatrelvir is a protease inhibitor, which is boosted with ritonavir to increase its concentrations. The protease enzyme is crucial for viral replication, and nirmatrelvir has shown to have antiviral activity against Coronavirus so far. The current recommendation for mild-moderate, nonhospitalized patients who are at a high risk of disease progression is to use Paxlovid as the first-line option. The commonly used regimens for Paxlovid is nirmatrelvir 300 mg with ritonavir 100 mg, per oral dose twice daily for 5 days to be started within 5 days of the onset of symptoms as early as possible in patients ≥12 years of age and ≥40 kg of weight (level of evidence AIIa). It is not known if courses with shorter durations will be associated with resistance or less efficacy. The major side effects and drug interactions are due to ritonavir. Dysgeusia, diarrhea, hypertension, and myalgia are the major side effects of Paxlovid. Dose adjustment is needed with moderate renal impairment and is not recommended in patients with eGFR <30 ml/min. For patients with mild or moderate hepatic impairment, it should be used with caution; however, for severe hepatic impairment, e.g., Child-Pugh class C, it is not recommended [10].

The pregnant and lactating women were not included in this trial; however, ritonavir is used safely in pregnant patients with HIV. Hence, on the basis of animal data and the mechanism of action of both nirmatrelvir and ritonavir, the final decision was made to administer Paxlovid to pregnant women if the benefits outweigh the risks. In vitro and in vivo data have suggested that ritonavir-boosted nirmatrelvir (Paxlovid) should remain active against the Omicron variant as well. However, due to its side effects and significant drug interactions, it might not be the safe drug of choice for Omicron patients [10].

### 4.2. Clinical Trial Data

EPIC-HR (NCT04960202) is the most important multinational, randomized trial for oral Paxlovid twice a day for 5 days in mild-moderate nonhospitalized COVID-19 patients. Unvaccinated patients with symptom onset within 5 days and at risk of progression of illness were included, and patients using medications which are strong inducers of CYP3A4 or are dependent on CYP3A4 for clearance were excluded from the trial. The primary outcome was hospitalization or mortality through Day 28 in patients who received the drug within 3 days of symptom onset. 2246 patients were enrolled in the trial. The demographic profile of participants consisted of 51% males and 72% white race, and the mean age was 46 years. SARS-CoV-2 antibodies were negative in 47% of patients and Paxlovid was administered to 66% of patients within 3 days of symptom onset.

1379 participants were randomized within 3 days of symptom onset to the Paxlovid or placebo group. There were 5 hospitalizations out of 697 participants (0.72%) in the Paxlovid group as compared to 44 out of 682 (6.45%) hospitalizations in the placebo group. In 2085 participants randomized within 5 days of symptoms, 8 out of 1039 (0.8%) in the Paxlovid group and 66 out of 1046 (6.3%) participants in the placebo group had hospitalizations due to COVID-19-related illnesses. There were no deaths in the Paxlovid group as compared to 12 deaths in the placebo group. The EPIC-HR trial has shown to reduce the risk of hospitalization or mortality by 88%, (*P* < 0.0001) as compared to placebo in confirmed cases of SARS-CoV-2 who were not hospitalized. The efficacy of Paxlovid is quite comparable to that of sotrovimab and remdesivir and greater than that of molnupiravir [10].

### 4.3. Molnupiravir

It is an oral prodrug of a ribonucleoside, beta-D-N4-hydroxycytidine (NHC). It has antiviral activity against RNA viruses including SARS-CoV-2. The viral RNA-dependent RNA polymerase uptakes the NHC and leads to lethal viral mutagenesis. As there could be a potential risk of genotoxicity with molnupiravir, in vivo rodent studies were performed, and the FDA has concluded a low risk of genotoxicity. The dose of molnupiravir is 800 mg twice a day for 5 days per oral. It should be administered to adults ages >18 years within 5 days of symptom onset ONLY if other options cannot be used (class IIa). The main side effects of molnupiravir are diarrhea, nausea, and dizziness. Molnupiravir is not recommended in pregnancy due to the risk of teratogenicity and fetal toxicity [11]. However, pregnant patients with more than 10 weeks of gestation who are at risk of severe disease can be administered molnupiravir after detailed discussion and documentation of the risks and benefits of treatment. For lactating mothers on molnupiravir, breast feeding is to be avoided; the breast milk is to be pumped and discarded while on therapy and after 4 days of the final dose. Molnupiravir is to be avoided in children <18 years of age as data are not available and there is a potential risk of cartilage and bone damage with molnupiravir. Men of reproductive age who are sexually active should abstain from sex or use strict contraception for 3 months after therapy. In vaccinated people, there are no data on the use of molnupiravir [11].

### 4.4. Clinical Trial Data

The MOVE-OUT, a multinational, randomized trial in phase 3, showed a 30% reduction in hospitalization and mortality with molnupiravir as compared to placebo. There were 1433 participants with a median age of 43 years, and 17% participants were >60 years old. 48% of participants experienced symptom onset within ≤3 days. The demographic profile consisted of 49% males, 57% whites, 50% Hispanic/Latino, and 5% African American. 74% of participants had a body mass index ≥30, and 16% participants had diabetes [11].

48 participants out of 709 (6.8%) in the molnupiravir group and 68 out of 699 (9.7%) in the placebo group had hospitalizations or deaths by day 29. There was a 30% relative risk reduction with molnupiravir, *P* = 0.0218. Total deaths in the molnupiravir group were 1 and in the placebo group were 9 deaths. As the efficacy of molnupiravir is lower, the current recommendations to use molnupiravir are to use it only when other medications, i.e., Paxlovid, sotrovimab, and remdesivir cannot be administered or are unavailable. For the Omicron variant, the data on the use of molnupiravir are limited. However, it is expected to be effective against the Omicron variant too.

### 4.5. Sotrovimab

Bamlanivimab plus etesevimab, casirivimab plus imdevimab, and sotrovimab are various monoclonal antibodies which received emergency use authorization from FDA for use in mild-to-moderate, nonhospitalized cases to COVID-19 who were at risk of progression of illness and were found to reduce the risk of mortality and hospitalization by 70%–85% in comparison to the placebo [15]. Bamlanivimab plus etesevimab and casirivimab plus imdevimab are ineffective against Omicron; however, sotrovimab is found to be effective against Omicron VOC in vitro studies.

The dose of sotrovimab is 500 mg intravenous infusion, a single dose to be given within 10 days of symptom onset. It is to be administered in patients ≥12 years of age and ≥40 kg of weight, residing in areas prevalent for the Omicron variant (class AIIa). As the major side effect of sotrovimab is hypersensitivity reactions including anaphylaxis, it should be administered in a healthcare setting, where anaphylaxis can be managed [15].

### 4.6. Clinical Trial Data

The data from the COMET-ICE phase 3 trial, which consisted of participants with mild-moderate COVID-19, at risk of progressing to serious illness and >18 years old who presented within 5 days of symptom onset. The end point of hospitalization or death was seen in 3 participants out of 291 (1%) in the sotrovimab group, vs 21 participants out of 292 (7%) in the placebo group. There was a 6% absolute reduction and an 85% relative risk reduction (*P* = 0.002) in hospitalizations and deaths with sotrovimab as compared to placebo.

### 4.7. Remdesivir

Remdesivir has been approved by the FDA for hospitalized patients and is given as an IV infusion for 3 consecutive days in a dose of 200 mg IV on day 1, followed by 100 mg IV once daily on day 2 and day 3, to be administered within 7 days of symptom onset in patients ≥12 years of age and ≥40 kg of weight (class BIIa). As remdesivir can lead to hypersensitivity reactions, so it should be administered in settings where anaphylaxis can be managed. The FDA has approved remdesivir for hospitalized patients only, and use in nonhospitalized patients would be an off-label indication [16].

### 4.8. Clinical Trial Data

The PINETREE trial was a randomized placebo-controlled trial, where nonhospitalized participants were administered remdesivir for 3 consecutive days within 7 days of symptom onset. However, the trial was discontinued due to administrative reasons. The end point event of hospitalization or death was seen in 2 participants out of 279 (0.7%) in the remdesivir group and 15 participants out of 283 (5.3%) in the placebo group, leading to a 4.6% absolute risk reduction and an 87% reduction in relative risk of hospitalization and deaths in patients who received remdesivir (*P* = 0.008) [16]. In vitro and in vivo data suggest that remdesivir is effective against Omicron VOC; however, the intravenous route of administration does not make it the first-line therapy. Remdesivir is a good option to be used if Paxlovid and sotrovimab are unavailable or cannot be administered.

## 5. Recommendations

There are no clinical trials comparing the efficacy of the abovementioned 4 therapies with each other nor there are any data on the combination of two or more therapies. The current recommendation for mild-moderate, nonhospitalized patients are to use Paxlovid as the first-line option in nonhospitalized, high-risk patients. If it is not available or cannot be administered due to drug interactions, then the next best choice is sotrovimab. The third choice is remdesivir if sotrovimab is also not available, and molnupiravir is to be given if the other three options are not available or cannot be administered. However, to select the treatment option for a specific patient, various factors need to be considered such as the efficacy of the drug, its availability of drug, the set-up to administer parenteral drugs, patients taking medications which can have drug-drug interactions, and the local prevalence of Omicron [17]. All the SARS-CoV-2 therapeutic drugs have been evaluated initially in people who were not vaccinated and who were not hospitalized but who had a risk of progression to severe illness as unvaccinated patients or those who have not yet developed an adequate immune response to the vaccine are at substantial risk of progression to severe disease [17].

### 5.1. Vaccines against Omicron Variants

Though COVID-19 vaccines are helpful in preventing severe COVID illness, ICU admissions, and mortality, the initial studies for the Omicron variant with live or pseudovirus suggested a significant reduction in neutralizing activity. Eventually, the study with sera from fully vaccinated recipients of Pfizer or AstraZeneca after 5 months of complete vaccination as well as convalescent sera of COVID-positive patients 6–12 months after infection showed no inhibition for Omicron. However, there was a neutralizing activity against Omicron which was 6- to 23-fold lesser than against the delta variant with sera of recipients of a Pfizer booster shot and recipients of vaccination who had been previously infected with COVID. This study also concluded that Omicron escapes most of the monoclonal antibodies used for treatment for previous COVID strains [3, 4, 7, 18].

Various studies with neutralization of sera after mRNA vaccines against various COVID variants showed a significant reduction of neutralization for the Omicron variant. Wang et al. [18] used a focus reduction neutralization test (FRNT) to detect the neutralizing activity of 2 to 6 weeks postvaccination sera of 88 fully vaccinated recipients after the second shot. They found minimal impact on the neutralizing activity of sera with the virus 614G strain with the D614G substitution. The progenitor 614D reference virus which is close to the strain used to make mRNA vaccines neutralizing activity was better than other strains of viruses. The neutralizing antibody titers were slightly reduced with the Alpha variant (B.1.1.7); however, greater reductions but <4-fold as compared to the reference 614D strain were seen with gamma (P.1), delta (B.1.617.2 and AY.4.2), epsilon (B.1.427/B.1.429), zeta (P.2), eta (B.1.525), iota (B.1.526/B.1.526.1), lambda (C.37), and B.1.617.3 variants. More than a 4-fold reduction in titers was seen with the beta (B.1.351), theta (P.3), kappa (B.1.617.1), Mu (B.1.621), and Omicron (B.1.1.529) variants. Omicron showed a 38-fold reduction as compared to reference 614D strain and failed to get neutralized with antibody sera [18]. They also compared neutralization of various strains with prebooster sera collected on the day of the booster shot to 2 to 6 weeks postbooster vaccination sera. The neutralizing titers with booster sera were much higher than prebooster sera for the 614D strain. For the Omicron variant, the titers were higher with booster sera as compared to prebooster sera. Overall, there was a 34–38-fold reduction of neutralizing activity with prebooster sera and a 19-fold reduction with booster sera for the Omicron variant. This probably explains the reason for worldwide breakthrough infections with the Omicron variant with waning immunity [19, 20]. In an ongoing phase 2-3 trail, 50-*μ*g bivalent mRNA-1273.214 vaccine containing 25 *μ*g each of ancestral Wuhan-Hu-1 and Omicron B.1.1.529 mRNAs was compared to a 50-*μ*g mRNA-1273 booster. The neutralizing antibody response against Omicron elicited by bivalent vaccine was superior to that with mRNA-1273 without any safety concerns [2, 19].

### 5.2. Efficacy of Monoclonal Antibodies against Omicron

Monoclonal antibody REGN10933 (casirivimab) lost neutralizing activity; however, REGN10987 (imdevimab) retained neutralizing activity against BA.2.12.1, BA.4, and BA.5 as seen on live-virus focus reduction neutralization testing (FRNT). The combination of casirivimab and imdevimab could also inhibit these isolates. Similarly, COV2-2196 (tixagevimab) had neutralizing activity against BA.2.12.1 but not against BA.4 or BA.5. COV2-2130 (cilgavimab) neutralized BA.2.12.1, BA.4, and BA.5. The combination of tixagevimab and cilgavimab inhibited BA.2.12.1, BA.4, and BA.5. The precursor of sotrovimab (S309) lost inhibitory property against BA.2.12.1, BA.4, and BA.5. Of the FDA-approved monoclonal antibodies, only LY-CoV1404 (bebtelovimab) efficiently neutralized BA.2.12.1, BA.4, and BA.5, similar to those for the ancestral strain [21]. Nevertheless, current evidence is still insufficient to draw meaningful conclusions regarding the utility of injected SARS-CoV-2-neutralizing mAbs [22], whose widespread use is hampered by at least three limitations: first, loss of neutralization capacity toward emerging variants [23]; second, health system sustainability because of the costs of these products administered in gram doses and produced in inexpensive mammalian expression systems; and third, the risk for antibody-dependent enhancement (ADE) of the infection [24]. In addition, recent data indicate that after the systemic infusion of a neutralizing mAb, SARS-CoV-2 is still present in the nasal turbinates [25], where the virus initially harbors and from where it spreads, making intranasal and aerosol treatments particularly attractive [26]. Small, single-domain V_H_H nanobodies, specific for SARS-CoV-2, were proposed for topical use (i.e., by inhalation) as an alternative to systemic mAbs [27, 28]. However, being nanobodies of camelid origin, they require sophisticated humanization procedures to avoid immunogenic responses, potentially hampering their full development [28]. Because of these limitations, the use of small human antibodies has emerged in the format of single-chain variable fragments (scFvs) that share several advantages with nanobodies with fewer risks.

### 5.3. Single-Chain Fragment Variable (scFv) Antibodies against Omicron

Despite the continuous efforts to curb and contain the emerging strains of Coronavirus and Omicron VOC, there is a persistent need for effective and efficient antibodies to neutralize the emerging strains. As the newer strains of Coronavirus are more adaptable and spread rapidly, it is imperative to anticipate newer mutations in the future and synthesize highly specific antibodies that are humanized, have better tissue penetrance and higher efficacy with minimal side effects, and can be produced in a short duration of time. Considering all these qualities, for future considerations, we propose the development and use of scFv antibodies against Omicron that possess all the abovementioned said qualities and can be readily produced when needed. Furthermore, since the ScFv antibodies lack the Fc fragment of the antibody component, they are free of side effects as seen with monoclonal antibodies. It has been reported that scFv antibodies generated by using phage display technology have high neutralizing effect against SARS-CoV infection [29, 30]. IgY-scFv has been extensively studied for the treatment of various respiratory viruses prior to COVID-19 [31–33]. Minenkova et al. [34] described engineered human antibody fragments (scFvs), which are extremely effective at neutralizing SARS-CoV-2 variants including Omicron [34]. Because of their high stability and efficacy in preclinical models, intranasal or aerosol delivery of scFv antibodies represents a promising approach for halting SARS-CoV-2 infection at an early stage, regardless of vaccination status [34]. Unlike conventional methods of antibody production, ribosome display is a rapid technique that can generate a huge repertoire of scFv monoclonal antibodies against SARS-COV-2. Highly specific recombinant antibodies can be isolated from libraries by manipulating the stringency of binding between antibodies to the target. Recently, our laboratory has refined a rapid ribosome display method to develop panels of scFvs against Ebola virus glycoprotein (GP) and Zika virus envelope (E) proteins [35–37]. This method is inexpensive, rapid, and can be used to quickly develop repertoires of high-affinity human scFv antibodies against SARS-CoV-2 Delta and Omicron variant spike proteins, which may offer an alternative solution in providing a large number of highly specific antibodies in combating COVID-19 in different application scenarios. Advantages of our approach include (1) recombinant antibodies can be generated at very little cost when compared to conventional monoclonal antibodies and (2) the platform allows for a rapid method to generate new antibodies based on predicted epitope sequences of escape mutants or novel emerging viral strains.

## 6. Conclusion

The highly transmissible Omicron variant is usually responsible for mild-to-moderate infection with lesser severity than the delta variant. The increased transmissibility is due to the mutations in the spike protein and the receptor-binding domain (RBD). Omicron is susceptible to oral antiviral drugs such as Paxlovid (nirmatrelvir/ritonavir), remdesivir, sotrovimab, and molnupiravir. The potency of sotrovimab is reduced to a 3-fold against Omicron. The current recommendation for mild-moderate, nonhospitalized patients are to use Paxlovid as the first-line option, followed by sotrovimab, followed by remdesivir. Molnupiravir is to be given if the other three options are not available or cannot be administered. Vaccine efficacy is reduced against the Omicron variant; however, booster doses with mRNA vaccines are found to be helpful in preventing the infection against the Omicron variant. The development of high-affinity human scFv antibodies using a ribosome display antibodies library may prove a resourceful arsenal containing therapeutic antibodies for passive therapy, antibodies for developing serological test kits, and variable heavy chain and light chain sequences for mutated viruses.

## Figures and Tables

**Table 1:** Structural and clinical characteristics of delta and Omicron variants.

Characteristics	Delta (B.1.617.2)	Omicron (B.1.1.529)	References
Number of mutations	17	30 or more	[1]
Transmissibility defined by effective (instantaneous) reproduction number	High	3.19 times greater than that of delta (95% confidence interval: 2.82–3.61)	[5]
Reinfection	Breakthrough infections seen	More common than delta but less severe illness	[6,7]
Vaccine efficacy (VE)			[6]
3 dose of VE at 14–60 days	93.7%	71.6%	
3 dose of VE after 60 days	86%	47.4%	
Clinical profile	Can be severe	Mild-to-moderate	
(1) Incubation period	4-5 days	3 days	[7]
(2) Contagiousness	One person can infect 2-3 persons	One person can infect 6 persons	[8]
(3) Duration of illness	14 days usually	Less than 10 days usually	[9]
(4) Severity of illness	More severe	Mild-to-moderate	[10]
(5) Anosmia and dysgeusia	More common	Rare	[11]
Diagnosis by RT-PCR (NAAT) tests	Not impacted	Not impacted except for Thermo Fischer TaqPath due to deletion of position 69-70 in spike protein	[1]
Severity of cases	Very severe	40–70% less severe than delta	[12, 13]
Hospitalisation rate	Higher rate as compared to Omicron	53% reduction compared to delta	[14]
ICU admissions	Higher as compared to Omicron	74% reduction compared to delta	[14]
Need for invasive ventilation	Higher as compared to Omicron	91% reduction compared to delta	[14]
Mortality	Higher rate as compared to Omicron	Very rare, almost zero as compared to delta	[14]
Length of hospital stay	Longer duration	3.4 days shorter or 69.6% reduced as compared to delta	[14]
Therapeutics and drugs efficacy			[9–11, 15–17]
(1) Remdesivir	Effective	Effective	
(2) Ritonavir-boosted nirmatrelvir	Effective	Effective	
(3) Molnupiravir	Effective	Effective	
(4) Sotrovimab (VIR-7831)	Effective	3-fold reduced efficacy	
(5) REGEN-COV™	Effective	Not effective	
(6) Bamlanivimab with etesevimab	Effective	Not Effective	

## Data Availability

The data used to support the findings of this study are included within the article.
